# The Diagnostic and Prognostic Value of Dengue Non-Structural 1 Antigen Detection in a Hyper-Endemic Region in Indonesia

**DOI:** 10.1371/journal.pone.0080891

**Published:** 2013-11-19

**Authors:** Herman Kosasih, Bachti Alisjahbana, Susana Widjaja, Nurhayati, Quirijn de Mast, Ida Parwati, Patrick J. Blair, Timothy H. Burgess, Andre van der Ven, Maya Williams

**Affiliations:** 1 Health Research Unit, Faculty of Medicine, Padjadjaran University, Bandung, Indonesia; 2 Internal Medicine Department, Hasan Sadikin Hospital, Bandung, Indonesia; 3 Viral Diseases Program, U.S. Naval Medical Research Unit 2, Jakarta, Indonesia; 4 Department of Internal Medicine, Radboud University Nijmegen Medical Centre, The Netherlands; 5 Clinical Pathology Department, Hasan Sadikin Hospital, Faculty of Medicine, Padjadjaran University Bandung, Indonesia; Duke-National University of Singapore Graduate Medical School, Singapore

## Abstract

As dengue fever is undifferentiated from other febrile illnesses in the tropics and the clinical course is unpredictable, early diagnosis is important. Several commercial assays to detect dengue NS1 antigen have been developed; however, their performances vary and data is lacking from hyper-endemic areas where all four serotypes of dengue are equally represented. To assess the sensitivity of the Bio-Rad platelia Dengue NS1 antigen assay according to virus serotype, immune status, gender, and parameters of severe disease, acute sera from 220 individuals with confirmed dengue and 55 individuals with a non-dengue febrile illness were tested using the Bio-Rad platelia Dengue NS1 antigen assay. The overall sensitivity of the NS1 ELISA was 46.8% and the specificity was 100%. The sensitivity in primary infections was significantly higher than in secondary infections (100% vs. 35.7%). In secondary infections, the sensitivity of NS1 detection was highest in DENV-3 (47.1%), followed by DENV-1 (40.9%), DENV-2 (30%) and DENV-4 (27%) infections. NS1 was less frequently detected in sera with high titers of HI antibodies or in acute samples from patients whose pre-illness sera showed neutralizing antibodies to more than one serotype. The detection of NS1 was higher in females, severe cases, and individuals with lower platelet counts (<100,000/mm^3^). While the overall sensitivity of this NS1 ELISA is poor, our data suggest that in secondary infections, detection may be predictive of a more severe illness.

## Introduction

Dengue fever (DF) is a major public health problem with 50 million annual cases worldwide. It has been reported in more than 100 countries and continues to spread to previously unaffected regions[1]. DF is caused by dengue viruses (DENV), which consist of four serotypes: DENV-1, DENV-2, DENV-3 and DENV-4. Infection with any serotype usually results in asymptomatic infection or mild non-specific febrile illness, but in a subset of patients, severe disease develops, characterized by a transient capillary leakage syndrome (dengue hemorrhagic fever/ DHF)[2]. This unpredictable disease course and the need to differentiate DF from other causes of fever make an early and sensitive diagnosis of acute dengue virus infection important. 

Non-structural 1 protein (NS1) is encoded by the virus and secreted in a soluble form[3]. Several commercial assays to detect dengue NS1 have been developed recently[4]. Advantages of NS1 tests are: NS1 is detected early in disease, several days prior to the appearance of anti-dengue IgM antibodies[5], and the test is inexpensive, easy, and fast. Moreover, NS1 levels early in dengue disease have been correlated with disease severity [6], suggesting that NS1 tests may also have prognostic value. 

Previous studies evaluating the diagnostic value of NS1 antigen assays have had varying results [4,7] (Table S1). Several factors may account for this: proportion of primary vs. secondary infections [4,8-11], timing of sample collection[4,7,8,12,13], infecting serotype[4,7,8,13,14], viremia levels [6,13,15,16] and severity of illness[6,7,15]. A low sensitivity has been reported in secondary infections possibly due to immune-complex formation of NS1 and pre-existing antibodies [17]. This may especially be important as the proportion of secondary infections is expected to rise worldwide [18]. Although studies evaluating the NS1 assays were conducted in hyper-endemic areas of Southeast Asia or South America, in several studies, all four dengue serotypes were not represented equally [4,7,8,10,13,15,16,19,20] or evaluated [11,21]. In others, the percentage of primary infections was too high to represent the situation in hyper-endemic areas [9,11,12,15,20]. Additionally, only a few studies evaluated the association between NS1 detection and clinical severity [4,6,7,15,21]. The objectives of the study were: 1) Determine diagnostic performance of the Bio-Rad Platelia Dengue NS1 antigen assay in a region where all dengue serotypes circulate year round and clinical severity could be evaluated 2) Assess the sensitivity of this assay in secondary cases according to pre-illness and acute dengue immune status 3) Determine the sensitivity of this assay according to clinical severity. 

## Materials and Methods

### Ethics Statement

All samples were obtained after written informed consent through studies approved by the Institutional Review Board of NAMRU#2 (N2.2006.0001, N2.2004.0010, N2.2004.0008) and at the National Institute of Health Research and Development, Ministry of Health, Indonesia (KS.02.01.2.1.2776, KS.02.01.2.1.3461 and KS.02.01.2.1.2336) in compliance with all U.S. Federal Regulations governing the protection of human subjects.

### Sample characteristics

Acute sera from archived and fresh samples of 220 confirmed dengue cases and 55 non-dengue febrile illness cases were used. Samples were from previously published studies[22,23]. Study one was a prospective dengue cohort study of ~3,000 factory workers in two factories in Bandung, Indonesia from 2000-2004 and 2006-2009. Volunteers participated in serosurveys every three months and visited factory clinics when they experienced fever. Study two was a community dengue study where 15-20 people living near a confirmed dengue case were observed for two weeks when a dengue case was confirmed at the study hospital. Volunteers in study one and two were hospitalized when dengue infection was confirmed. Study three was a hantavirus surveillance study at two hospitals in Bandung, Indonesia. As clinical manifestations between dengue and hantavirus are indistinguishable, samples were screened for evidence of dengue infection. In all studies, acute sera were collected when volunteers presented at clinics or hospitals, and convalescent samples were collected during hospital discharge or 7-10 days after acute sample collection. Paired samples were tested for evidence of dengue infection as described below. Complete blood counts to detect thrombocytopenia and hematocrit measurements to look for hemoconcentration as one indicator of plasma leakage were taken daily for hospitalized patients and upon indication for outpatients. Ultrasonography to detect plasma leakage was performed on hospitalized patients in study one and two. 

### Case Definitions

A dengue infection was confirmed when 1. DENV RNA was detected by RT-PCR or 2. For RT-PCR negative cases, when all of the following criteria were met: IgM positive, increasing IgG titers and a four-fold rise in hemagglutination inhibition (HI) titers. A primary or secondary dengue infection was determined by the absence or presence of IgG antibodies in the acute sample[24]. Non-dengue infection was confirmed when dengue RT-PCR was negative, and no IgM antibodies, increase in IgG titers or four-fold increase of HI antibody titers was detected. Infecting serotypes were determined by RT-PCR and neutralizing antibodies to each serotype in pre-illness sera were measured by plaque reduction neutralization test (PRNT). Dengue cases were classified as DF, DHF, and dengue shock syndrome (DSS) according to 1999 WHO guidelines[25]. Cases of DF with hemorrhagic manifestations formed a separate group (DF+HM).

### Laboratory tests

Acute serum samples were assayed for DEN virus RNA by RT-PCR[26]. Acute and convalescent sera were tested for dengue IgM, IgG and HI antibodies. Dengue IgM and IgG were detected by capture and indirect ELISA respectively according to the manufacturer’s instructions (FOCUS Technologies, CA, USA). HI tests were performed as previously described[27]. Acute samples were tested with the NS1 Platelia antigen capture ELISA according to the manufacturer’s instructions (BioRad, CA, USA). PRNT was conducted on pre-illness sera collected during the serosurvey in study one from 2000-2004. Three 10-fold serum dilutions were made starting at 1:10 and diluted samples were assayed as described previously[28]. The dilution that produced an 80% reduction in plaque count compared with the negative control sample was determined by probit analysis using SPSS (SPSS, Chicago, IL). The virus strains used in the assays were isolated from DEN patients in Thailand (16001 DENV-1, 16682 DENV-2, and 16562 DENV-3) and Indonesia (1036 DENV-4).

### Statistical analysis

Data were entered into Microsoft Access. Statistical analysis was performed using STATA version 9 (STATA Corporation, TX). Categorical variables between two groups were compared with a chi-square test. A p value of less than 0.05 was considered significant.

## Results

### Overall and serotype specific sensitivity of the NS1 test

Samples from 275 volunteers were used, 220 confirmed dengue cases and 55 non-dengue cases. Dengue infection was confirmed by both virological and serological evidence in 195 (88.6%) cases and by serological evidence alone in 25 (11.4%) cases. Table 1 summarizes the characteristics, WHO clinical category, virological and serological data for these cases. The overall sensitivity of the assay was 46.8% (95% CI: 40.2-53.3) and the specificity was 100% (55/55) (Table 2). The sensitivity was 50.3% (95% CI: 43.3-57.3) in samples positive by RT-PCR, and 20% (95% CI: 4.3-53.7) in samples negative by RT-PCR. The sensitivity was 100% (34/34) in primary infections and 35.7% (95% CI: 28.7-42.7) in secondary infections (p=0.00). In secondary infections, no significant (p=0.38) difference was found between fresh and archived specimens (38.9%, 95%CI: 28.8-49 vs.32.6%, 95%CI: 23-42.2); however, the sensitivity by serotype varied. The highest sensitivity was observed for DENV-3 (47.1%, 95% CI: 35.2-58.9), followed by DENV-1 (40.9%, 95% CI: 20.4-61.4), DENV-2 (30%, 95% CI: 13.6-46.4) and DENV-4 (27%, 95% CI: 12.7-41.3). This difference in sensitivity was significant when comparing DENV-3 to DENV-4 (p=0.045.).

**Table 1:** Specimen characteristics.

**Source of Specimens**	**Study 1**	**Study 2**	**Study 3**	**Total**
Non-dengue	51	4	0	55
Acute dengue	125	27	68	220
Mean age in years (range)	36.5 (21-53)	19.5 (4-47)	24.4 (12-50)	30.7 (4-53)
Sex Ratio (male:female)	3.3:1	1:1.1	1.4:1	2.1:1
Outpatients	72	8	0	80
Inpatients	53	19	68	140
Mean day after illness onset (range)	2.7 (1-7)	2 (1-7)	5.1 (2-7)	3.5 (1-7)
Clinical diagnosis (%)				
*dengue fever (DF)*	84	9	33	126 (57.3)
*DF with hemorrhagic manifestations*	8	2	22	32 (14.5)
*dengue hemorrhagic fever grade I*	18	6	5	29 (13.2)
*dengue hemorrhagic fever grade II*	15	8	8	31 (14.1)
*dengue shock syndrome*	0	2	0	2 (1)
Infecting serotype (%)				
DENV-1	23	6	6	35 (15.9)
DENV-2	19	5	18	42 (19.1)
DENV-3	35	6	40	81 (36.8)
DENV-4	32	1	4	37 (16.8)
Unknown	16	9	0	25 (11.4)
Type of infection (%)				
Primary	16	5	13	34 (15.4)
Secondary	109	22	51	182 (82.7)
Unknown	0	0	4	4 (1.8)

**Table 2:** Sensitivity of the NS1 antigen test according to infection status.

	**Infecting serotypes**					
**Type of infection**	**DENV-1**	**DENV-2**	**DENV-3**	**DENV-4**	Unknown*	**TOTAL**
Primary	13/13(100)	11/11(100)	10/10(100)	0/0(0)	0/0(0)	34/34(100)^1^
Secondary	9/22 (40.9)	9/30 (30)	32/68(47.1)^2^	10/37(27)^2^	5/25(20)	65/182(35.7)^1^
Unknown^#^	0/0(0)	1/1(100)	3/3(100)	0/0(0)	0/0(0)	4/4(100)
Total	22/35(62.9)	21/42(50)	45/81(55.6)	10/37(27)	5/25(20)	103/220(46.8)

* PCR negative, ^#^ IgG results not available,

1 statistically significant difference (p=0.00) ^2^statistically significant difference (p= 0.04)

### NS1 sensitivity according to dengue immune status

To determine if the sensitivity of the NS1 test varied by dengue immune status, dengue HI titers were determined on all acute samples and PRNTs were performed on pre-illness (one to two months prior to dengue infection) sera from the 47 patients enrolled in the first phase of study one. The sensitivity was significantly (p<0.05) lower in acute samples with high HI titers (≥1280) compared to samples with lower or undetectable titers (Table 3A). Among the 47 samples tested by PRNT, neutralizing antibodies to more than one serotype were detected in 32 cases and NS1 was only positive in one acute specimen, giving a sensitivity of 3.1%. In contrast, in patients with pre-illness neutralizing antibodies to only one serotype, the sensitivity of the NS1 test was 60% (9/15) (Table 3B). Among these nine NS1 positive cases, eight had pre-illness neutralizing antibodies to DENV-2 and one to DENV-1, whereas among the six negative cases, four had neutralizing antibodies to DENV-1, one to DENV-3 and one to DENV-2. The infecting serotypes in eight of the nine positive cases were DENV-1 (2), DENV-3 (2) and DENV-4 (4).

**Table 3:** Sensitivity of the NS1 antigen test according to dengue immune status.

**A. Acute specimen HI titer (n=179)**	
**HI titer**	**Sensitivity**
< 10	26/32(81.3%)^1^
10 - 80	29/72 (40.3%)
160 - 640	18/40 (45%)
≥1280	7/35 (22%)^1^
B. Presence of pre-illness neutralizing antibodies* (n=47)	
**Number of serotypes**	**Sensitivity**
1	9/15^4^ (60%)
more than 1	1/32^4^ (3.1)

1 significantly different from the other groups

*plaque reduction neutralizing antibodies (80%)

4 significantly different from each other

### NS1 sensitivity according to disease severity and gender

**Figure 1:**
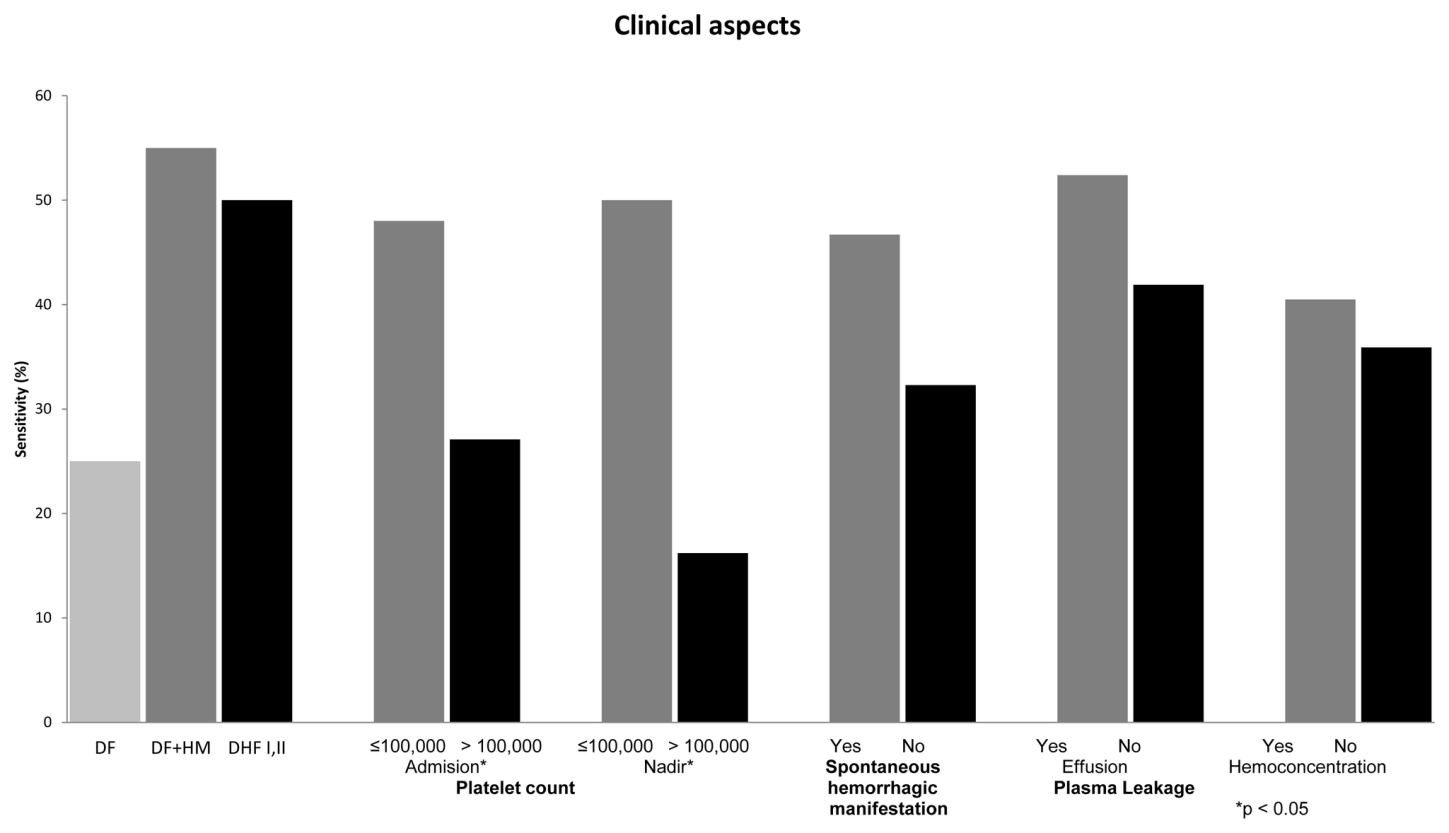
Sensitivity of the NS1 antigen test according to diagnosis category and clinical variables in secondary dengue cases.

**Figure 2:**
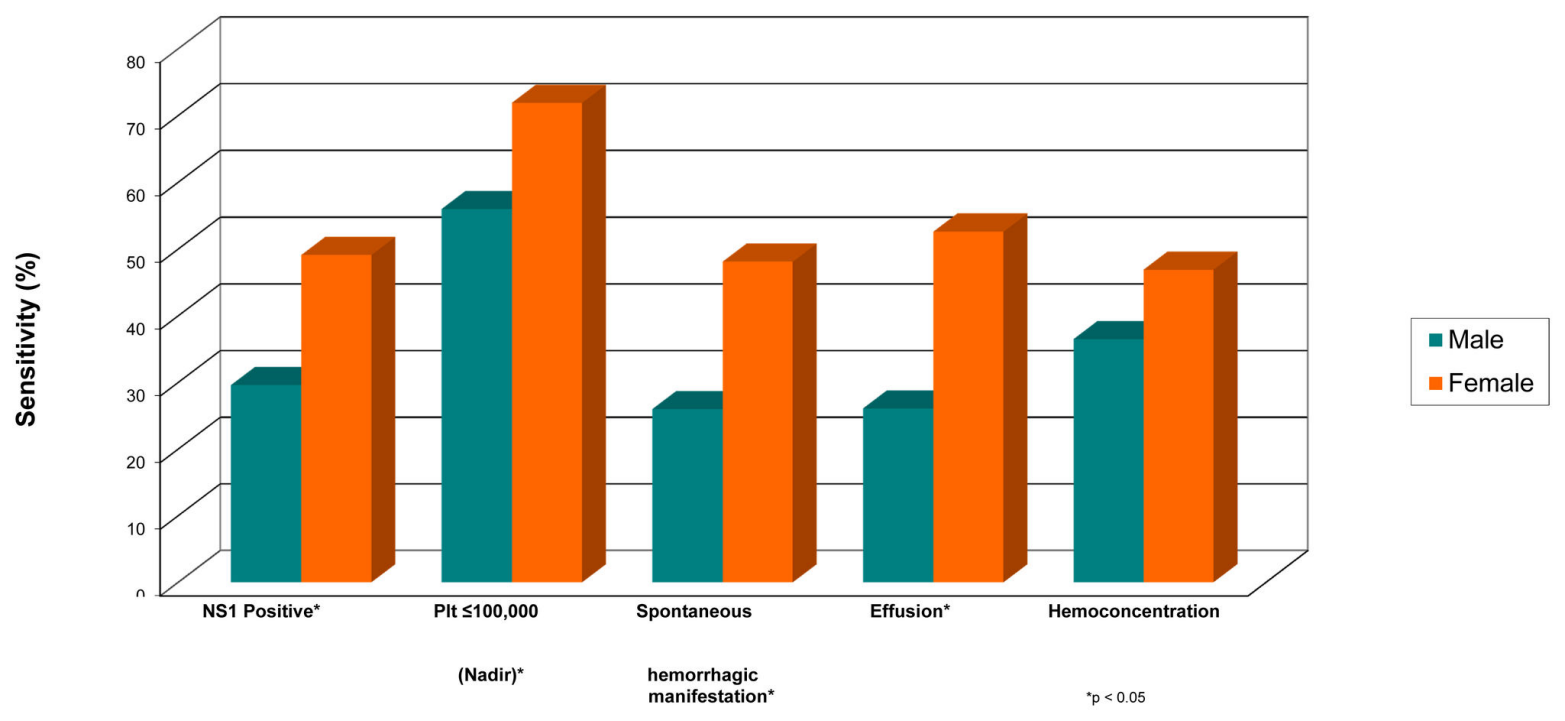
The sensitivity of the NS1 antigen test according to gender and clinical variables in secondary infections.

As the sensitivity of the NS1 assay was 100% in primary infections, only secondary infections were included in this analysis. The sensitivity among DF cases (25%, 95% CI: 16.7-33.3) was significantly lower compared to DF+HM cases (55%, 95% CI: 33.2-76.8) and DHF I and DHF II cases (50%, CI: 36.9-63.1). Additionally, the sensitivity was higher in patients with platelet counts below 100,000/mm^3^ at presentation (48% vs. 27.1%, p=0.00) or at time of platelet nadir (50% vs.16.2%, p=0.00). A higher sensitivity was also found in cases with spontaneous hemorrhagic manifestations, hemoconcentration or effusion, but these differences were not significant (Figure 1). Data from a subset of patients enrolled in study one and two in which patients were identified early, revealed that the mean and range interval between the detection of NS1 and thrombocytopenia among 28 patients was 1.8 (0-5) days, whereas the time between the detection of NS1 and evidence of plasma leakage among 17 DHF patients was 3.3 (1-5) days. The sensitivity of the NS1 assay was significantly higher in females than males with values of 49.1% (95% CI: 36.1-62.1) and 29.6% (95% CI: 21.6-37.6) respectively (p=0.01). This gender difference remained present when analysis was restricted to those with manifestations of more severe disease (Figure 2). 

## Discussion

 NS1 tests are increasingly being used as a rapid and inexpensive diagnostic tool for acute DF. We found that its sensitivity in a dengue hyper-endemic area (Indonesia) where most infections are secondary was low with an overall sensitivity of 46.8%. In samples from primary infections, the sensitivity was 100% indicating that it is a good diagnostic tool for patients from non-endemic areas, such as in travelers[29]. Our findings are also in line with reports from other countries that found a low sensitivity in secondary infections [4,13,20]. Apart from primary or secondary infection status, our data also suggest that sensitivity was affected by dengue immune status, infecting dengue serotype, gender and severity of illness.

Our data demonstrated poor performance of the NS1 test in acute sera with high titers of HI dengue antibodies. Further analysis showed that NS1 antigen was undetectable in nearly all acute sera from patients with pre-illness neutralizing antibodies to more than one serotype, but was detectable in more than half of those with only one serotype. These findings are in line with the hypothesis that immune-complexes are formed in secondary infections and high levels of antibodies capture and bind soluble NS1 (sNS1) resulting in a reduction of circulating NS1[17]. Therefore, the use of a NS1 assay in areas where post-secondary dengue infection is common [30,31] has limited value. The serotype of a previous dengue infection may also influence the sensitivity of NS1 detection in secondary infections as we found the sensitivity was significantly higher in acute samples from patients with pre-illness neutralizing antibodies to DENV-2 (88.9%) compared to DENV-1 (11.1%). It has been reported that in comparison to DENV-1 and DENV-3, DENV-2 infections result in much lower concentrations of sNS1[32]. Thus, during primary DENV-2 infections, the amount of sNS1 produced may be unable to generate sufficient NS1 antibodies to form immune-complexes with sNS1 in secondary infections. The influence of pre-existing antibodies to other flaviviruses that may circulate in the region such as Japanese encephalitis as a result of natural infection or vaccination may also affect the sensitivity of NS1 and should be considered. The data presented here are consistent with previous studies that report a higher sensitivity of the NS1 test in DENV-3 and DENV-1 infections compared to DENV-2 and DENV-4 infections [12,14]. As the specimens used in this study included all DENV serotypes, the overall sensitivity was not affected by a predominating serotype as has been reported elsewhere [7]. 

Our study also demonstrated that in secondary cases, the sensitivity is better in patients with more severe illness. The sensitivity of the test in the DF group was significantly lower compared to the other groups (DF+HM or DHF). DSS cases were excluded from this analysis, as the number of DSS cases was too small (n=2). The association between NS1 and clinical severity has been studied before with discordant results[4,6,7,15,21]. The differences might be due to inadequate number of cases[21] or the application of different clinical category guidelines [4]. NS1 sensitivity was also significantly higher in patients with platelet counts below 100,000/mm^3^ measured at presentation only or nadir platelet counts below 100,000/mm^3^ if serial measurements were done. A positive NS1 result in secondary infections may therefore be a predictor for low platelet count. Although not significantly different, sensitivity tended to be higher in cases with spontaneous bleeding, effusion and hemoconcentration. The association between NS1 and clinical severity is thought to be related to the cross-reactivity of anti-dengue NS1 antibodies with platelets and endothelial cells, and the role of NS1 in the occurrence of vascular leakage through complement and cytokine responses [33,34]. Recent findings have also shown that NS1 positivity is correlated with higher viremia or antigenemia which was found in more severe cases[13,15]. 

The overall sensitivity of the NS1 test and its sensitivity in secondary infections in our study is lower than what has previously been published. There are several possible reasons for this difference. This and other studies have demonstrated that the sensitivity of NS1 in secondary infections is much lower than in primary infections. As a consequence, the overall sensitivity will be affected by the proportion of primary or secondary infections in the sample set used. Compared to studies conducted in other endemic countries, our study has a low proportion of primary infections. Our study also included outpatients (36.4%). As has previously been reported and is found in this study, NS1 sensitivity is associated with disease severity. Therefore, outpatients in our study, which were mostly mild cases, may have contributed to the lower NS1 sensitivity. Additionally, we found that among secondary cases, post-secondary (≥2) cases (cases with pre-illness neutralizing antibodies to more than one serotype) had lower sensitivities than secondary cases with pre-illness neutralizing antibodies to only one serotype. Based upon the data from the 47 subjects that were tested for pre-illness neutralizing antibodies and the mean age of our volunteers, we suspect that many of the cases in our study were post-secondary infections. Finally, the proportion of secondary infections caused by DENV-2 and DENV-4, serotypes with lower sensitivities in our study, was higher than in other studies. 

The strengths of this study are: 1) work was conducted in a setting where all dengue serotypes were equally distributed and the ratio between primary and secondary infections reflected the regional epidemiology 2) accurate clinical outputs were available as patients were monitored closely 3) this study provides a wealth of laboratory data to assess the correlation between NS1 detection and serotype distribution, and dengue immune status including serotype specific pre-existing immunity, which have not been reported elsewhere. This study has also several limitations. First, male participants were more predominant than females. However, despite fewer samples from females, the detection of NS1 was significantly higher in females than males. Second, ultrasonography was not performed on a subset of volunteers. As a consequence, several DF+HM cases might be more accurately included in the DHF group. However, the numbers are probably small as daily hemoconcentration was performed on all individuals. Third, PRNT was only conducted on pre-illness sera from 47 patients. Although, the results showed a strong association between sensitivity and the presence of neutralizing antibodies to DENV-2, further study is needed.

In conclusion, the NS1 test is not recommended as a standalone diagnostic test for dengue infection in regions where secondary infections predominate. However, as the sensitivity and specificity in acute specimens from primary infections is excellent, use of the test may prove of value to travelers returning from dengue endemic areas. Furthermore, as the sensitivity in secondary cases is associated with clinical severity, NS1 may be useful to predict severe disease. However, further studies with larger sample sizes and in other geographic regions are needed to confirm this association.

## Supporting Information

Table S1 **NS1 Study Results.**
(DOC)
