# Supplementary material for: The Diagnostic and Prognostic Value of Dengue Non-Structural 1 Antigen Detection in a Hyper-Endemic Region in Indonesia
Source: PLoS One. 2013 Nov 19;8(11):e80891. doi: 10.1371/journal.pone.0080891 (PMC3834322; doi:10.1371/journal.pone.0080891)
Supplement: Table S1 — NS1 Study Results. (DOC) [file pone.0080891.s001.doc]

Table S1. NS1 Study Results

|  |  |  | Sensitivity^1^ and proportion of specimens (both in %) by category | | | | | | | | | |
| --- | --- | --- | --- | --- | --- | --- | --- | --- | --- | --- | --- | --- |
|  |  |  | Immune status | | Serotype | | | | | Day of specimen collection | Severity of Illness | Viremia (pfu/ml) |
| Author (Year/Country) | N (Gold standard) | Overall sensitivity^1^ | 1^o^ case | 2^o^ case | DENV1 | DENV2 | DENV3 | DENV4 | Unknown |
| Osorio (2010/Colombia) | 212 (isolation, RT-PCR, serology) | 70.8% | 83-90^2^ (29.4) | 40-50^2^ (70.6) | 80-90^2^ (27.4) | 50-60^2^ (20) | 80-90^2^ (43.7) | 40-50^2^ (8.9) | exact data not available | day 1-3:70-80^2^ (NA), day 4-7 60-70^2^ (NA) | **Non Severe:** 70-80^2^ (75.7),  **Severe:** 50-60^2^ (24.3) | NA |
| Libraty (2002/Thailand) | 32 (RT-PCR) | 81.3% | 0 | 81.3 (100) | 0 | 81.3 (100) | 0 | 0 | 0 | NA | **DF:** 71(43.8),  **DHF I,II,III:** 89(56.3) | associated with NS1 level which is higher in DHF than DF |
| Guzman (2010/multiple) | 1284 (isolation, RT-PCR, serology) | 66% (34-76%) | ND | ND | 87 (32.3) | 63 (20) | 82 (11.1) | 79 (3.7) | 43.4 (37) | **Latin America^2^**, day2: 40-50, day3: 60-70, day 4: 60-80, day 5: ND, day 6: ND; **Southeast Asia^2^,** day2: 70-80, day3: 70-80, day 4: 60-70, day 5: 50-60, day 6: 30-40 | **Latin America**: DF 41, DHF 68; **Southeast Asia**: DF: 70,  DHF: 68 | NA |
| Hang (2009/Vietnam) | 125 (RT-PCR or serology) | 83.2% | 95.8 (19) | 78.5 (74) | 98 (50.4) | 55 (20) | 96 (25) | ND (2.4) | ND (11.2) | day 0-3 90.6 (60), day 4-6 70 (40) | NA | Significantly higher in NS1 positive patients |
| Najioullah (2011/Martinique) | 264 (RT-PCR) | 61.2% | 85 (28.6) | 48 (71.4) | 0 | 61.2 (100) | 0 | 0 | 0 | day 1-5: 64.4 (NA), day 6: 37.9 (NA), day 7-8: 61.9 (NA) | NA | NA |
| Chaterji (2011/Singapore) | 154 (isolation, RT-PCR) | 80.5% | 94.7 (48.7) | 67.1 (51.3) | 79.6 (35.1) | 73.9 (29.9) | 87 (35.1) | 0 | 0 | day 1: 64.1 (25.3), day 2: 82.8 (37.7), day 3: 89.5 (37), An increased sensitivity for secondary infection between days 0 and 3 | NA | NA |
| Thomas (2010/Martinique) | 67 (RT-PCR) | 67.1% | 69.2 (74.2) | 61.5 (25.7) | 0 | 61.9 (30) | 0 | 69.4 (70) | 0 | day 1-3: 69.4 (70), day 4: 61.9 (30) | The proportion of NS1 was prolonged in secondary or severe infections | NS1+ correlated with viral loads |
| Blacksell (2008/Laos) | 38 (RT-PCR, serology) | 63.2% | 75 (10.5) | 60.1 (89.5) | 77.8  (23.7) | 60  (13.2) | 0  (5.3) | 66.7 (23.7) | 61.5 (34.2) | day 1-4: 76.9 (16.5)  day 5-7: 75 (83.5) | NA | NA |
| Lima (2010/Brazil) | 220 (isolation, RT-PCR + serology) | 83.6% | 95 (74.1) | 71.4 (25.9) | 98  (22.7) | 90 (22.7) | 86.2 (26.4) | 0 | 64.5 (28.2) | day 1-4: >80 (N/A), day 5-7: 75 (N/A) | NA | NA |
| Lapphra (2008/Thailand) | 171 (isolation, RT-PCR, serology) | 63.2% | 76.9 (7.6) | 64.5 (92.4) | NA | NA | NA | NA | N/A | Not associate with days of fever (63.2-75.7) | **DF:** 65.1 (88.3**); DHF:** 68.4 (11.7) | NA |
| Kumarasamy (2009/Malaysia) | 213 (isolation, RT-PCR) | 93.4% | 97.3 (86.4) | 70 (13.6) | NA | NA | NA | NA | 0 | NA | NA | NA |
| Bessoff (2008/Puerto Rico) | 208 (RT-PCR or isolation) | 83.2% | 98.3 (27.9) | 77.3 (72.1) | 92.9  (26.9) | 82.2 (21.6) | 86.5 (25) | 70.9 (26.4) | 0 | day 1: 70-80, day 2: 80-100, day 3: 70-80, day 4: 70-80, day 5: 80-100 | NA | Not related to NS1 positvity |
| Tricou (2010/Vietnam) | 245 (RT-PCR) | 61.6% | 80.3 (27.3) | 55.1 (72.7) | NA  (56.3) | NA (37.6) | NA (6.6) | 0 | 0 | ≤day 3: 60.9 (63.7), > day 3: 62.9 (36.3) | NA | NS1 + samples significantly higher viremia than NS1- |
| Dussart (2008/French Guaina) | 272 (isolation, RT-PCR) | 87.4% (serotyped samples) | 96.3 (40.1) | 73 (59.9) | 90.9  (12.1) | 85.7 (15.4) | 87.1 (37.1) | 87 (16.9) | 60 (18.4) | day 0-3 89.7 (72.1) | NA | NA |
| Duong (2011/Cambodia) | 243 (isolation, RT-PCR, NS1, serology) | 57.5% | 87.5 (14.5) | 53.5 (85.5) | 80  (21) | 60 (4) | 63.6 (46.5) | 53.3 (5.8) | 8.5 (22.7) | day 1-2 81 (8.8), day 3 60-80 (23), day 4 60-80(32.2), day 5 40-60 (20.1), day 6 20-40 (11.3), day 7-8 <20 (4.6) | **DF** 72.3 (39), **DHF/DSS** 40.2 (35.8), **Indeterminate** NA (28) | NS1+ significantly higher in subjects with high viremia |

^1^ When a study compared the sensitivities between two or more kits, the one listed is the result from ELISA Platelia NS1.

^2^ Proportions are obtained from figures which do not include the exact percentages.
